# Bacterial Metabolites Produced Under Iron Limitation Kill Pinewood Nematode and Attract *Caenorhabditis elegans*

**DOI:** 10.3389/fmicb.2019.02166

**Published:** 2019-09-19

**Authors:** Diogo Neves Proença, Thomas Heine, Christoph H. R. Senges, Julia E. Bandow, Paula V. Morais, Dirk Tischler

**Affiliations:** ^1^Department of Life Sciences and Laboratory of Environmental Microbiology of CEMMPRE, University of Coimbra, Coimbra, Portugal; ^2^Environmental Microbiology, TU Bergakademie Freiberg, Freiberg, Germany; ^3^Applied Microbiology, Faculty of Biology and Biotechnology, Ruhr University Bochum, Bochum, Germany; ^4^Microbial Biotechnology, Faculty of Biology and Biotechnology, Ruhr University Bochum, Bochum, Germany

**Keywords:** metallophores, siderophore, nematode, *Erwinia*, *Rouxiella*, *Caenorhabditis elegans*, *Bursaphelenchus xylophilus*, secondary metabolites

## Abstract

Pine Wilt Disease (PWD) is caused by *Bursaphelenchus xylophilus*, the pinewood nematode, and affects several species of pine trees worldwide. The ecosystem of the *Pinus pinaster* trees was investigated as a source of bacteria producing metabolites affecting this ecosystem: *P. pinaster* trees as target-plant, nematode as disease effector and its insect-vector as shuttle. For example, metals and metal-carrying compounds contribute to the complex tree-ecosystems. This work aimed to detect novel secondary metabolites like metallophores and related molecules produced under iron limitation by PWD-associated bacteria and to test their activity on nematodes. After screening 357 bacterial strains from Portugal and United States, two promising metallophore-producing strains *Erwinia* sp. A41C3 and *Rouxiella* sp. Arv20#4.1 were chosen and investigated in more detail. The genomes of these strains were sequenced, analyzed, and used to detect genetic potential for secondary metabolite production. A combinatorial approach of liquid chromatography-coupled tandem mass spectrometry (LC-MS) linked to molecular networking was used to describe these compounds. Two major metabolites were detected by HPLC analyses and described. One HPLC fraction of strain Arv20#4.1 showed to be a hydroxamate-type siderophore with higher affinity for chelation of Cu. The HPLC fraction of strain A41C3 with highest metal affinity showed to be a catecholate-type siderophore with higher affinity for chelation of Fe. LC-MS allowed the identification of several desferrioxamines from strain Arv20#4.1, in special desferrioxamine E, but no hit was obtained in case of strain A41C3 which might indicate that it is something new. Bacteria and their culture supernatants showed ability to attract *C. elegans*. HPLC fractions of those supernatant-extracts of *Erwinia* strain A41C3, enriched with secondary metabolites such as siderophores, were able to kill pinewood nematode. These results suggest that metabolites secreted under iron limitation have potential to biocontrol *B. xylophilus* and for management of Pine Wilt Disease.

## Introduction

The pine wilt disease (PWD) is one of the most devastating diseases of forests pine trees in the world, with tremendous ecological, environmental, and economic damage. Native to North America (United States and Canada), it has spread to Asia and Europe (Proença et al., 2017b). The only known causal agent is the nematode *Bursaphelenchus xylophilus*, the pinewood nematode (PWN; Nickle et al., 1981). PWN is transferred when an insect vector feeds on young shoots of pine trees and, if the tree is susceptible, develops the disease and starts the PWN-insect life cycle (Proença et al., 2017b). The ecosystem in which the PWD is developed is rather complex and comprises next to above mentioned tree-nematode-insect also adjacent microorganisms. Respectively, the endophytic microbiome of pine trees was recently characterized (Proença et al., 2017a; Alves et al., 2018), and the bacteria carried by PWN (Proença et al., 2010, 2014; Vicente et al., 2012) and insect vector were studied (Alves et al., 2016, 2018). Especially, the microbiome of the nematode seems directly linked to the tree-wilting (Alves et al., 2018). Further, the tree and nematode microbiome differ significant with respect to genus/species level. In addition, some of the relevant and more tree-related bacteria are endophytic opportunistic, living in soil and may enter the tree through the roots (Proença et al., 2017a). They are believed to be plant growth promoting rhizobacteria (PGPR) or do at least affect and thus interact with plants. They might not only promote the growth of the trees but also can control plant pests (Niranjan Raj et al., 2005). PGPR produce various compounds as for example small organic acids, phytohormones, antibiotics and siderophores among others. Metabolites like organic acids and siderophores interfere with metal or metalloid abundance and mobility in rhizosphere and surrounding environment. Especially the availability of metals, like iron (Fe^3+^ or Fe^2+^), was determined to be crucial within plant-microbe-nematode interactions (Ramamoorthy et al., 2001). It was reported that under iron limitation not lipopolysaccharides but siderophores as pseudobactin induce systemic resistance towards plant pests (Saikia et al., 2005; Romera et al., 2019). This response seems to be complementary to other defense mechanisms in dependence on the environmental conditions.

Metals are essential for life, acting as cofactors of enzymes and participating in central biological processes, as for example oxygen binding, activation and transport, photosynthesis, and within electron transport chains (Fones and Preston, 2013). The oxidation state of each metal is dependent on the environmental conditions that in turn results in its bioavailability for living organisms. Iron and copper are important metals for biological processes and its availability in soil influences plant growth as well as microorganisms (beneficial and pathogenic) (Złoch et al., 2016). Thus the redox environment and availability of certain metals and metalloids defines the ecosystem, especially in the rhizosphere and its vicinity (Zobel et al., 2005; McNear, 2013). In order to acquire certain metals and metalloids, organisms like microbes (e.g., the PGPR) and plants secrete metallophores which support the mobilization of poorly soluble metals (in case of Fe^3+^ designated as siderophores) (Hider and Kong, 2010).

Metallophores have distinct biochemical characteristics and are divided into various families, i.e., hydroxamates, catecholates, carboxylates, and mixed types (Hider and Kong, 2010; Saha et al., 2013). Different types of siderophores have different metal binding capabilities and are often not restricted to iron but can scavenge different metals like copper, zinc, vanadium, gallium, and cadmium from the environment (Mehnert et al., 2017; Wiche et al., 2017; Schwabe et al., 2018). There are several biotechnological applications of siderophores, for example, enhancing of plant growth, acting as biological control agents (BCA), to perform bioremediation of environments contaminated with metals, to recover rare earth elements, and to act as antibiotic carriers or inhibitors of metalloenzymes (Ahmed and Holmström, 2014; Kurth et al., 2016). Plants are not able to assimilate iron in its predominant form in soil, ferric ion or Fe^3+^, and produce phytosiderophores that mobilize Fe^3+^ and are being taken up as Fe^3+^-complexes (Ahmed and Holmström, 2014). Bacterial siderophores have been shown to be important in this interplay, increasing uptake of iron into tissues of the model plant *Arabidopsis thaliana* and improved plant growth (Vansuyt et al., 2007). On the other hand, bacterial siderophores are suggested to be pathogenicity factors because they compete for iron, limiting iron uptake by the plant (Gamalero and Glick, 2011; Wilson et al., 2016).

Overall it can be stated that metal limitation is stress for all the organisms of such an ecosystem described herein and the response siderophore production, and secretion can have various outcomes for the individuals. And as described above this can also affect the PWD-causing nematode and needs, therefore, to be investigated. The effects of siderophores towards the model nematode *Caenorhabditis elegans* is controversial. It was shown that bacterial siderophores from pseudomonads chelate iron and lead to death of *C. elegans* (Kirienko et al., 2013; Kang et al., 2018). However, siderophores like enterobactin caused an increase in iron levels in mitochondria, promoting the growth of *C. elegans* (Qi and Han, 2018).

Considering the large interest in the metabolic arsenal of environmental bacteria with biotechnological potential, the present work focused on the siderophores produced by wood colonizing and/or endophytic bacteria from the rhizosphere, where a complex community comprising plants and nematodes among others, interacts. Therefore, the present work aims to identify the siderophores produced by endophytic bacteria isolated associated with *Pinus* trees, in order to understand the role of bacterial siderophores using the model nematode *C. elegans* and verify their nematicidal activity towards the PWN. Two strains producing distinct siderophore patterns were chosen as model systems to investigate siderophore production, metal binding properties, and the biochemical properties of the siderophores, as well as their pathogenicity against nematodes.

## Materials and Methods

### Bacterial Strains, Culture Conditions and Biochemical Characterization

Two hundred and seventy-one bacterial strains isolated as wood colonizers/endophytes of *P. pinaster* trees from Portugal (Proença et al., 2017a), 48 bacterial strains carried by PWN from Portugal (Proença et al., 2010), and 38 bacterial strains carried by PWN from United States (Proença et al., 2014) were evaluated for their production of siderophores on Iron-CAS agar plates (Schwyn and Neilands, 1987). The screening for bacterial endophytes and bacteria carried by PWN nematode from Portugal were re-evaluated in this study alongside with bacterial strains carried by PWN from United States for a confident result. The two best bacterial siderophores-producers candidates, with highest orange halo (positive for siderophore secretion) and lack of information of siderophores-producing species, strains Arv20#4.1 and A41C3, were selected for further production and characterization.

These two strains were grown in tubes with 5 ml of M9 media, supplemented with glucose (4% w/v), succinic acid (4 g/l), and Mg/Ca/B1/Goodie Mix (Esuola et al., 2016) for seven days. To investigate siderophore production and bacterial growth, the supernatant of each strain was obtained at days 0, 3, 5, and 7 and the OD_600_ was determined at the same time points.

The phenotypes of strains Arv20#4.1 and A41C3 were analyzed focusing on the solubilization of phosphate and zinc, production of siderophores, and hydrolyzing activities including protease (casein), lipase (Tween 20, 40, 60 and 80), cellulase, and chitinase activities (Naveed et al., 2014). The ability to oxidize different carbon sources was assessed using Biolog GN2 MicroPlates incubated at 30°C. The results were recorded daily for up to 72 h using a MicroPlate reader (Tecan Infinite M200).

### Phylogenetic Analysis

The strains Arv20#4.1 and A41C3 were grown on R2A agar media at 30°C for 48 h and its genomic DNA was extracted using the E.Z.N.A. Bacterial DNA Kit (Omega Bio-Tek, Norcross, GA, United States) according to the manufacturer’s instructions.

These strains were phylogenetically identified by 16S rRNA gene sequencing using primers 27F and 1525R as previously described (Rainey et al., 1992). All obtained sequences from bacterial isolates were compared with sequences available in the EMBL/GenBank database using BLASTN network services (Altschul et al., 1997) and with sequences available on the Eztaxon-e server^1^ (Kim et al., 2012). Sequences were aligned within the SINA alignment service (Pruesse et al., 2012). Sequences were also checked for chimeric artifacts by using Mallard software (Ashelford et al., 2006). Sequences were included in 16S rRNA-based Living Tree Project (LTP) release 115 database^2^ by parsimony implemented in the ARB software package version 5.5 (Ludwig et al., 2004). Phylogenetic dendrograms of sequences from this study and closest reference sequences were constructed by the Randomized Axelerated Maximum Likelihood (RAxML) method with GTRGAMMA model (Stamatakis, 2006) included in the ARB software (Ludwig et al., 2004).

### Genome Sequencing, Annotation and Siderophore Mining

The draft genome sequencing of strains Arv20#4.1 and A41C3 was generated on an Illumina platform (MiSeq) as described previously (Retamal-Morales et al., 2018). To this end standard Illumina protocols and kits were employed. The extracted genomic DNA (see above) was used to prepare a genomic library using the NexteraXT DNA library kit. A quality check was performed using an Agilent Bioanalyzer 2100. The paired-end libraries obtained were sequenced employing v3-v4 chemistry on the MiSeq platform. The reads obtained were analyzed by applying FastQC, TrimGalore including the Phred20 quality filter, and SPAdes 3.5 for read assembly by using the GALAXY webtool^3^. The quality of the assembled genomes was accessed by means of Quast (Gurevich et al., 2013). The assembled genomes were annotated by using automatic pipelines of NCBI Prokaryotic Genomes Automatic Annotation Pipeline (PGAP^4^), RAST (Aziz et al., 2008) and JGI servers (Huntemann et al., 2015; Chen et al., 2016, 2017).

Both annotated draft genomes were subjected to secondary metabolite gene cluster analysis by using the platform antiSMASH 3.0 (Weber et al., 2015), with specific interest for siderophores. Therefore, a BLAST analysis was performed for all protein sequences codified by each gene from siderophore gene cluster by using NCBI (Altschul et al., 1997) and UniProtKB/Swissprot and Uni-ProtKB/TrEMBL (Schneider et al., 2004) databases.

### Determination of Siderophore Production

Supernatants obtained from cultures of strains Arv20#4.1 (bacterium carried by PWN from United States) and A41C3 (endophytic bacterium of *P. pinaster* from Portugal) were assessed for the siderophore activities by a modified CAS assay and a modified Csáky test (Esuola et al., 2016; Mehnert et al., 2017). EDTA and desferrioxamine B (DFOB) were used as positive controls. The modified CAS assay was used to determine Fe, Ga, Cu, V, and Al chelating compounds in comparison to the same control reagents as for the Csáky test (Mehnert et al., 2017). Fe-, Ga-, Al-CAS solutions were prepared as follows (100 ml): 1.5 ml of 1 mM FeCl_3_ × 6H_2_O (in 10 mM HCl) or 1.5 ml of 1 mM Ga_2_(SO_4_)_3_ or 1.5 ml of 1 mM AlCl_3_ × 6 H_2_O was added to 7.5 ml of 2 mM aqueous CAS solution. Six ml of a 10 mM hexadecyltrimethylammonium bromide (CTAB) solution was added slowly. Subsequently MES buffer was added (10.67 g MES in 80 ml H_2_O with a pH of 5.6 by adjusting with 50% KOH). The mixture obtained was filled to a final volume of 100 ml with H_2_O. Cu-CAS solution was prepared as follows (100 ml): 2 ml of 10 mM CuCl_2_, 10.5 ml of 2 mM aqueous CAS solution, 2.5 ml of ddH_2_O and 85 ml of MES buffer at pH 5.7. V-CAS solution was prepared as follows (100 ml): 1.5 ml of 5 mM VCl_3_, 7.5 ml of 2 mM aqueous CAS solution, 6 ml of 10 mM CTAB and 85 ml of MES buffer at pH 4.1. Supernatants were mixed with Fe-CAS, Al-CAS, or V-CAS solution in a ratio 1:1 or were mixed with Ga-CAS or Cu-CAS solutions in a ratio 1:5. 33 mM of EDTA (final concentration) was used as positive control and ddH_2_O was used as negative control. Absorbance was measured at 630 nm for Fe, Ga, and Al assays, at 590 nm for V assays, and at 582 nm for Cu assays, after 4 h of incubation (except 6 h for Al assay).

Catecholate siderophore production was estimated using the colorimetric Arnow assay (Arnow, 1937) with following modifications: 150 μl of supernatant, 150 μl of 0.5 N HCl, 150 μl of nitrite-molybdate (10 g sodium nitrite and 10 g sodium molybdate per 100 ml) and 150 μl of 1 N NaOH were added in this order. The absorbance was measured at 510 nm. The standard curve was based on 3,4-dihydroxybenzoic acid (DHBA) (Sigma).

Hydroxamate siderophore production was estimated using the colorimetric Atkin assay (Atkin et al., 1970). Briefly, 104 μl of supernatant was added to 52.5 μl of 2 N perchloric acid. From this mix, 47.5 μl were added to 47.5 μl of 10% sodium acetate. Then, 47.5 μl of 1% sulfanilic acid and 19 μl of 0.5% I_2_ and incubated at room temperature (RT) for 15 min. 19 μl of 0.1 N sodium thiosulfate and 19 μl of 0.6% α-naphthylamine were added consequently and incubated at RT for 45 min. The absorbance was measured at 562 nm. The standard curve was based on NH_2_OH (Sigma).

### Enrichment of Siderophores by HPLC

The strains Arv20#4.1 and A41C3 were selected to be grown in high volumes, 1 l of M9 medium mention above for each strain and supernatant was harvested by centrifugation at 10,000 rpm at 4°C for 1 h. The pH of each supernatant was measured. The siderophores in the supernatants of each strain were obtained by ion exchange-based solid phase extraction as follows: Amberlite XAD-4 (4 g/l) with Amberlite XAD-16 (4 g/l) activated with dH_2_O, followed by methanol and washed with dH_2_O, and incubate at 4°C for 3 days. Then, the supernatant was discarded and beads with siderophores were washed 3 times with 30 ml of dH_2_O, followed by shaking 3 times with 20 ml methanol at 65 rpm at room temperature. These volumes of methanol were evaporated in an evaporator system at low pressure to a final volume of 5 ml.

The siderophores were enriched by HPLC to a C18-RP column (1 ml; Thermo, Auqastar) equilibrated in 28% of methanol (buffer A) and 72% of ddH_2_O with 0.1% of TFA (buffer B) (Anke et al., 2017; Schwabe et al., 2018). The column elution was performed as described and after 8 min a linear gradient from 28 to 95% buffer A was performed to 12 min, buffer A concentration kept constant for 3 min before switching back to initial conditions (total time of elution 15 min at 1 ml/min flow rate). The eluted molecules were detected at 217 nm and 430 nm. The resulting 30 fractions were characterized for their Fe, Ga, Cu, V, and Al-CAS activities and hydroxamate and catecholate siderophores were quantified as mentioned in previous section.

### LC-MS/MS Analysis

Fractions from HPLC profiling with a significant CAS activity were analyzed by LC-MS/MS and fragment spectra were investigated by molecular networking. Samples were dried under reduced pressure and reconstituted in 1 ml of MS-grade methanol. To achieve a better solubility these samples were sonicated for 5 min. Mass spectrometric analysis was performed similarly as described earlier (Senges et al., 2018). Liquid chromatography (LC) was performed on a nanoACQUITY-UPLC system (Waters, Milford, MA, United States) with a Mixer Assy (Waters, zirc bead, inner cross section 1 mm, length 50 mm) and an AcquityUPLC HSS T3 column (Waters, pore size 100 Å, particle size 1.8 μm, inner cross section dimension 1 mm, length 100 mm). 4.7 μl of each sample was injected and separated by means of a H_2_O/acetonitril (ACN) gradient with 0.1% formic acid (FA) and a flow rate of 25 μl min^–1^ (Table 1).

**TABLE 1:** Gradient used for LC-MS/MS analysis.

**Time [min]**	**% ACN with 0.1% FA**
0.0	5.0
2.5	5.0
21.0	99.5
23.0	99.5
28.5	5.0
30.0	5.0

Subsequently, the mass spectrometry (MS) was performed with a Synapt G2-S HDMS^E^ system (Waters) with an ESI source operated in positive and negative mode and a TOF detector. Masses in the range of 50 to 3000 m/z were detected with 0.5 s per scan and leucine enkephalin injected as reference mass every 30 s. Mass spectrometry settings: lockspray capillary current 2.5 kV, capillary current 2.5 kV, cone voltage 30 V, source temperature 120°C, cone gas flow 60 l/h, flushing gas flow 550 l/h, with a temperature of 150°C. Fragmentation in MS/MS mode was conducted by collision induced diffraction with argon and a collision energy ramp of 10–25 V. Fragmentation was started when the intensity of a mass signal exceeded 6000 counts/s, finished after 6 s at max, and stopped prematurely if the intensity dropped below 6000 counts/s. Spectra were processed with MassLynx V4.1 SCN932 (Waters).

Using Proteowizard (ver. 3.0.9490) waters.raw-files were converted to.mzXML, with 32-bit binary encoding precision and peak picking. Spectra were uploaded to the UCSD GNPS FTP-server [ucsd.gnps.edu] and investigated using the METBOLOMICS-SNETS1 workflow with following parameters: parent mass tolerance 2 Da, ion tolerance 0.5 Da, minimal pairs cos 0.7, network topK 10, maximum connected component size 100, minimum matched peaks 6, minimum cluster size 2, run MSCluster. Data were visualized with Cytoscape (ver. 3.4.0). Network nodes corresponding to solvent background were excluded. Annotation of fragment spectra was aided by *in silico* fragmentation with MetFrag.

### Nematode Strains and Growth Conditions

The nematode wild type *Caenorhabditis elegans* N2, obtained from Caenorhabditis Genetic Center, Minneapolis, MN (United States) were propagated at 19.5°C on nematode growth agar medium (NGM) with *Escherichia coli* OP50 lawn (Brenner, 1974). After 3 days, nematodes were harvested by rinsing the plates with sterile M9 broth medium, transferred to 15 ml tubes in a total of 2 ml. It was added a fresh solution containing 0.5 ml of 2N NaOH and 1 ml of 3% sodium hypochlorite, followed by vortexing every 2 min with final duration of 10 min. The nematodes and eggs were centrifuged 1,300 g x 30 s at 22°C. Supernatant was removed and the nematodes and eggs were washed 2 times with sterile water. The eggs were transferred into new NGM plates and incubated at 19.5°C during 18 h - 24 h. The pinewood nematode *B. xylophilus* PtAS18, was maintained on agar plates containing *Botrytis cinerea* Pers. at 25°C (Fonseca et al., 2012).

### *C. elegans*-Bacterial Strains/Supernatants Attraction Assay

Chemotaxis assays were performed according to Akduman et al. (2018) with some modifications. Briefly, 10 μl of bacterial suspensions obtained after 24 h of growth in M9 broth media supplemented with iron, at 30°C at 130 rpm, were placed 0.5 cm away from the edge of a 6 cm NGM agar plate. Ten μl of *E. coli* OP50 were added on the opposing side. Idealizing an equilateral triangle, 10 μl containing approximately 30 J3 stage nematodes were placed on the remaining vertex, with all spots at the same distance.

In the same line, 10 μl of bacterial supernatants, obtained from bacterial growth as mentioned above, was used to perform the chemotaxis attraction. Moreover, the bacterial strains were also grown in M9 broth medium without supplementation of iron, and also its suspensions and its supernatants were tested in chemotaxis attraction of *C. elegans* N2.

Different combinations were performed to understand the attraction of the nematodes by different bacteria, its supernatants and if the influence of the presence of iron in M9 broth media also affects their attraction. M9 broth media with or without iron supplementation was also tested in chemotaxis assays. After 3h the number of nematodes were recorded in each test spot. All the assays were performed in triplicate.

### Nematodes Survival Assays

The bacterial strains and their supernatants mentioned above were used to evaluate their effect on *C. elegans* N2 viability. The nematodes were observed and scored at 24 h, 48 h, and 72 h for with a dissecting microscope. On the other hand, the nematicidal activity of bacterial strains, its supernatants and HPLC fractions were evaluated towards the *B. xylophilus* PtAS18 (mixed life stages: J4 and adults) and towards *C. elegans* N2 (mixed life stages: L4 and adults) according to Paiva et al. (2013) with little modifications. Briefly, the bacterial suspensions obtained were centrifuged (20 min, 4°C, 13,000 rpm), in order to drastically reduce the number of cells, without removing any growth product. To test the nematicidal activity, 500 μl of each bacterial supernatant, with less than 0.06 O.D.600 nm, were incubated with 30 disinfected nematodes, for 48 h at 25°C. The assays were performed in triplicate. Nematodes were disinfected by sequential washes in sodium hypochlorite 0.1% (one wash, 1 min at 4°C) and in 1 ml sterilized water (two washes, 3 min at 4°C), followed by centrifugation. Last water (100 μl) was inoculated on R2A for control of the disinfection efficiency. The number of dead nematodes was assessed under the stereoscopic microscope. Nematodes were considered dead when linearized and not able to recover after being transferred to water. The controls, nematodes in CAA, were incubated under the same conditions.

### Statistical Analysis

Chi-squared test was used to evaluate the significance of the differences in the chemotaxis of *C. elegans* with bacterial and supernatant samples. All differences were considered to be statistically significant for *p* < 0.05.

The significance of the differences, observed between the different nematicidal treatments towards *B. xylophilus* PtAS18 by using bacterial supernatants or HPLC fractions, was evaluated with two-way analysis of variance (ANOVA), using Bonferroni post-tests. The statistical analysis was performed with GraphPadPrism v5.0 for windows, GraphPad software, San Diego, CA, United States. All differences were considered to be statistically significant for *p* < 0.05.

### Accession Numbers

The Whole Genome Shotgun project of strain Arv20#4.1 and A41C3 have been deposited at ENA under the accession numbers RQVU00000000 and RQVV00000000, respectively. The 16S rRNA gene sequences of the isolates, have been previously deposited in the NCBI GenBank database under the accession numbers according KF214948 (Proença et al., 2014) and KJ654833 (Proença et al., 2017a). Accession links for metabolomic data sets are provided at: positive mode (http://gnps.ucsd.edu/ProteoSAFe/status.jsp?task=bbd4f668b6224bd381617001be917381); negative mode (http://gnps.ucsd.edu/ProteoSAFe/status.jsp?task=e3009a90a0954791ae9b747e727c6515).

## Results

### Siderophore Screening, Bacterial Phylogeny and Biochemical Characterization

In this study, 357 bacterial strains were tested and belonging to 52 genera according to 16S rRNA gene analysis (Proença et al., 2010, 2014, 2017a). Numerous bacterial strains were positive on CAS agar medium that serves to detect siderophores: 103 endophytic strains (38.0%), 39 strains carried by *B. xylophilus* from Portugal (81.2%) and 20 strains carried by *B. xylophilus* from United States (52.6%) (Supplementary Figure S1). Among those, two isolates showed a distinct siderophore production on CAS agar plates and have been chosen for further study: strains Arv20#4.1 and A41C3.

According to neighbor-joining and maximum-likelihood phylogenetic trees, the closest relatives to strain Arv20#4.1 were *Rouxiella chamberiensis* (99.6% sequence similarity), *R. silvae* (99.6%), and *R. badensis* (99.6%). *Erwinia endophytica* (98.1%), *Pantoea dispersa* (97.7%), and *E. psidii* (97.6%) were the closest relatives to strain A41C3. Thus, the best two bacterial isolates with higher biotechnological potential analyzed in this study belong to the family *Enterobateriaceae*, and were *Erwinia* sp. A41C3 (endophyte from Portuguese pines) and *Rouxiella* sp. Arv20#4.1 (bacterium isolated as carried by PWN from United States) (Supplementary Figure S2).

Biochemical characteristics of strains Arv20#4.1 and A41C3 are summarized in Table 2. Both strains were able to solubilize phosphate and zinc, produce siderophores, degrade Tween 60 and carboxymethyl cellulose (CMC), and produced catalases; but were negative for protease production, chitin, Tween 40 and Tween 80 degradation. Both strains oxidized 43 carbon sources in GN2 MicroPlate (and 6 more carbon sources were weakly oxidized). Only the strain A41C3 was able to oxidize m-inositol, β-hydroxybutyric acid, α-keto butyric acid, α-keto glutaric acid, sebatic acid, succinamic acid, L-pyroglutamic acid, γ-amino butyric acid in GN2 MicroPlates (Table 2).

**TABLE 2:** Differential characteristics between strains *Rouxiella* sp. Arv20#4.1 and *Erwinia* sp. A41C3.

**Characteristic**	**Arv20#4.1**	**A41C3**
Phosphate solubilization	W	+
Siderophores	+++	++
Tween 20	++	−
CMC degradation	+	−
Catalase	+	++
BIOLOG		
Tween 80	W	+
M-Inositol	−	+
D-Sorbitol	+	W
Turanose	W	+
Pyruvic Acid Methyl Ester	−	W
*Cis*-Aconitic acid	W	+
Formic acid	+	W
D-Glucosaminic acid	W	+
α-Hydroxybutyric acid	W	+
β-Hydroxybutyric acid	−	+
p-Hydroxy Phenylacetic acid	−	W
α-Keto butyric acid	−	+
α-Keto glutaric acid	−	+
α-Keto valeric acid	−	W
Malonic acid	W	+
Quinic acid	W	+
Sebatic acid	−	+
Bromosuccinic acid	W	+
Succinamic acid	−	+
L-Alaninamide	−	W
D-Alanine	+	W
L-Glutamic acid	W	+
Glycyl-L-aspartic acid	W	+
L-Proline	W	+
L-Pyroglutamic acid	−	+
L-Threonine	W	+
γ-Amino butyric acid	−	+
Urocanic acid	W	+

*Both strains were positive on solubilization of zinc, degradation of Tween 60; and negative on production of proteases, degradation of Tween 40, Tween 80 and chitin. Both strains oxidized Tween 40, N-acetyl-D-glucosamine, adonitol, L-arabinose, D-arabitol, D-cellobiose, erythritol, D-fructose, L-fucose, D-galactose, gentiobiose, α-D-glucose, m-inositol, α-D-lactose, lactulose, D-maltose, D-mannitol, D-mannose, D-melibiose, b-methyl-D-glucoside, D-psicose, D-raffinose, L-rhamnose, D-sorbitol, sucrose, D-trehalose, turanose, xylitol, pyruvic acid methyl ester, succinic acid mono-methyl-ester, acetic acid, *cis*-aconitic acid, citric acid, formic acid, D-galactonic acid, D-galacturonic acid, D-gluconic acid, D-glucosaminic acid, D-glucuronic acid, α-hydroxybutyric acid, β-hydroxybutyric acid, γ-hydroxybutyric acid, p-hydroxy phenylacetic acid, itaconic acid, α-keto butyric acid, α-keto glutaric acid, α-keto valeric acid, D,L-lactic acid, malonic acid, propionic acid, quinic acid, D-saccharic acid, sebatic acid, succinic acid, bromosuccinic acid, succinamic acid, glucuronamide, L-alaninamide, D-alanine, L-alanine, L-alanyl-glycine, L-asparagine, L-aspartic acid, L-glutamic acid, glycyl-L-aspartic acid, glycyl-L-glutamic acid, L-histidine, L-hydroxyproline, L-leucine, L-ornithine, L-phenylalanine, L-proline, L-pyroglutamic acid, D-serine, L-serine, L-threonine, D,L-carnitine, γ-amino butyric acid, urocanic acid, inosine, uridine, thymidine, phenyethylamine, putrescine, 2-aminoethanol, 2,3-butanediol, glycerol, D,L,α-glycerol phosphate, α-D-glucose-1-phosphate, D-glucose-6-phosphate. +, Positive; W, weakly positive; −, negative.*

### Genome Analysis Showed (Novel) Siderophore Gene Clusters

The draft genome sequence of strains Arv20#4.1 contained 29 contigs, totaling 5.37 Mbp in size with a mapped coverage of 547-fold of the genome. The G + C content was 53.0 mol%. The genome encoded 4,936 putative coding sequences (CDSs) (Table 3). The draft genome sequence of strain Arv20#4.1 contained loci for three ribosomal RNAs and 72 tRNAs (Table 3).

**TABLE 3:** General characteristics of the genome sequences of the strains *Rouxiella* sp. Arv20#4.1 and *Erwinia* sp. A41C3.

**Characteristic**	**Arv20-4-2**	**A41C3**
Sequence size	5,371,123	4,227,107
GC content (%)	53.0	51.6
N50	431,840	138,450
L50	4	8
Number of contigs (with PEGs)^a^	29	61
Number of Subsystems^a^	401	359
Number of RNAs^a^	74	64
Genome coverage^b^	547x	792x
Genes (total)^b^	5,021	4,023
CDSs (total)^b^	4,936	3,953
Genes (coding)^b^	4,867	3,765
CDSs (with protein)^b^	4,867	3,765
Genes (RNA)^b^	85	70
rRNAs (5S, 16S, 23S)^b^	1, 1, 1	5, 1, 1
Complete rRNAs^b^	1, 1, 1	5, 1, 1
tRNAs^b^	72	57
ncRNAs^b^	10	6
Pseudogenes (total)^b^	69	188
CDSs (without protein)^b^	69	188
Pseudogenes (ambiguous residues)^b^	0	0
Pseudogenes (frameshifted)^b^	25	86
Pseudogenes (incomplete)^b^	36	126
Pseudogenes (internal stop)^b^	20	36
Pseudogenes (multiple problems)^b^	11	54
CRISPR Arrays^b^	0	2

*^*a*^Data from RAST annotation; ^*b*^Data from PGAP from NCBI.*

In case of strain A41C3, the draft genome sequence contained 61 contigs, totaling 4.22 Mbp in size with a mapped coverage of 792-fold of the genome. The G + C content was 51.6 mol%. The genome encoded 3,953 putative CDSs (Table 3). The draft genome sequence of strain A41C3 contained loci for seven ribosomal RNAs and 57 tRNAs (Table 3). A general overview of the biological subsystems obtained using RAST for both strains is summarized in Table 4.

**TABLE 4:** Biological subsystem distribution of annotated genes in strains *Rouxiella* sp. Arv20#4.1 and *Erwinia* sp. A41C3.

**Code**	**Description**	**Arv20#4.2**	**A41C3**
A	Cofactors, vitamins, prosthetic groups, pigments	188	167
B	Cell wall and capsule	70	53
C	Virulence, disease and defense	61	42
D	Potassium metabolism	19	11
E	Miscellaneous	23	15
F	Phages, prophages, transposable elements, plasmids	79	12
G	Membrane transport	138	125
I	RNA metabolism	54	58
J	Nucleosides and nucleotides	101	96
K	Protein metabolism	221	202
L	Cell division and cell cycle	8	8
M	Motility and chemotaxis	14	0
N	Regulation and cell signaling	76	64
O	Secondary metabolism	5	4
P	DNA metabolism	78	84
Q	Fatty acids, lipids, and isoprenoids	71	55
R	Nitrogen metabolism	25	22
S	Dormancy and sporulation	3	2
T	Respiration	106	92
U	Stress response	96	73
V	Metabolism of aromatic compounds	14	12
W	Amino acids and derivatives	401	357
X	Sulfur metabolism	27	37
Y	Phosphorus metabolism	37	33
Z	Carbohydrates	382	243

By using the webtool antiSMASH, it was possible to identify the gene cluster for secondary metabolite, especially siderophore, production close to desferrioxamine, in particular the operon that is composed by decarboxylase, monooxygenase, and acetyltransferase in strain Arv20#4.1 (Table 5 and Supplementary Figure S3A). For strain A41C3, it was possible to identify multiple genes in the gene cluster for siderophore production (Table 6 and Supplementary Figure S3B). However, it was not possible to predict which siderophore is produced. There are several putative siderophore biosynthesis clusters identified by antiSMASH that could be attributed to strain A41C3 like as turnerbactin, griseobactin among others.

**TABLE 5:** Secondary metabolite clusters identified in strain *Rouxiella* sp. Arv20#4.1 with antiSMASH3.0.

**Cluster**	**Type**	**Start**	**Stop**	**Similarity to known cluster**	**MIBiG BGC-ID**
1	Hserlactone	87033	107683	–	–
2	Cf_fatty_acid	446284	467504	–	–
3	Arylpolyene	787129	821369	APE Ec biosynthetic gene cluster (68% of genes show similarity)	BGC0000836_c1
4	Cf_putative	25500	34695	Emulsan biosynthetic gene cluster (9% of genes show similarity)	BGC0000760_c1
5	Cf_fatty_acid-Cf_saccharide	24604	60942	Lipopolysaccharide biosynthetic gene cluster (18% of genes show similarity)	BGC0000776_c1
6	Cf_putative	27900	36897	–	–
7	Cf_putative	89005	95978	–	–
8	Cf_fatty_acid	165825	186739	–	–
9	Cf_saccharide	485089	506842	S-layer glycan biosynthetic gene cluster (20% of genes show similarity)	BGC0000794_c1
10	Cf_fatty_acid	532454	553725	–	–
11	Cf_putative	586070	593059	Polysaccharide B biosynthetic gene cluster (6% of genes show similarity)	BGC0001411_c1
12	Cf_putative	712377	716502	–	–
13	Cf_putative	103014	112356	–	–
14	Cf_putative	283336	295617	–	–
15	Siderophore	270474	282858	Desferrioxamine B biosynthetic gene cluster (60% of genes show similarity)	BGC0000941_c1
16	Cf_putative	164758	170600	–	–
17	Cf_saccharide	97264	131452	Capsular polysaccharide biosynthetic gene cluster (33% of genes show similarity)	BGC0000731_c1

*NRPS, Non-ribosomal peptide synthetase cluster; Cf, ClusterFinder.*

**TABLE 6:** Secondary metabolite clusters identified in strain *Erwinia* sp. A41C3 with antiSMASH3.0.

**Cluster**	**Type**	**Start**	**Stop**	**Similarity to known cluster**	**MIBiG BGC-ID**
1	Cf_putative	284735	289620	Polysaccharide B biosynthetic gene cluster (6% of genes show similarity)	BGC0001411_c1
2	Cf_putative	15697	24996	–	–
3	Cf_putative	68401	78206	–	–
4	Thiopeptide	210237	236598	O-antigen biosynthetic gene cluster (14% of genes show similarity)	BGC0000781_c1
5	Cf_fatty_acid	347964	368917	Svaricin biosynthetic gene cluster (6% of genes show similarity)	BGC0001382_c1
6	Cf_putative	72078	80802	–	–
7	Cf_fatty_acid	1	11819	–	–
8	Cf_putative	18987	28258	Emulsan biosynthetic gene cluster (9% of genes show similarity)	BGC0000760_c1
9	Cf_fatty_acid	1	7355	–	–
10	Cf_fatty_acid -Ladderane	35473	78034	–	–
11	Cf_fatty_acid	41264	62484	–	–
12	Cf_saccharide	70023	92437	–	–
13	Cf_saccharide	57635	93206	Lipopolysaccharide biosynthetic gene cluster (60% of genes show similarity)	BGC0000779_c1
14	Hserlactone	73942	94574	–	–
15	Cf_saccharide	30591	54347	–	–
16	Cf_saccharide	103519	141097	Stewartan biosynthetic gene cluster (85% of genes show similarity)	BGC0000763_c1
17	Cf_saccharide-NRPS	131937	194079	Turnerbactin biosynthetic gene cluster (30% of genes show similarity)	BGC0000451_c1
18	NRPS	271826	315746	–	–
19	Cf_putative	129079	138254	–	–
20	Cf_putative	93961	107030	–	–
21	Cf_putative	143921	152024	–	–
22	Butyrolactone	192718	198822	–	–

*NRPS, Non-ribosomal peptide synthetase cluster; Cf, ClusterFinder.*

### Production and Biochemical Characterization of Siderophores

**FIGURE 1:**
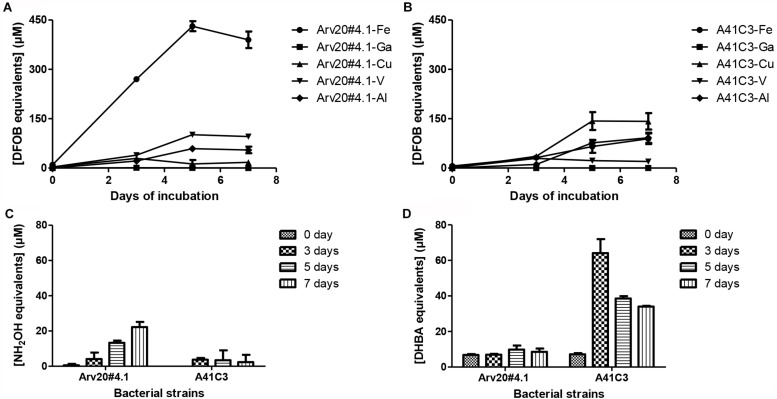
Bacterial production and characterization of siderophores with different metal ion affinities. The two bacterial strains produced siderophores with different metal binding affinities, Arv20#4.1 siderophores showed higher affinity to iron **(A)** and A41C3 siderophores to Cu **(B)**. The production of siderophores is dependent on the growth of the strains. The strain Arv20#4.1 predominantly produced hydroxamate-type siderophores **(C)**, strain A41C3 catecholate-type siderophores **(D)**.

Both bacterial strains reached maximum of optical density after 3 days of incubation in 10 ml tubes containing 5 ml of M9 modified medium. Siderophore production by both strains depended on days of cultivation, but both supernatants showed activity for hydroxamate and catecholate-type siderophores (Figure 1). Strain Arv20#4.1 produced mainly hydroxamate-type siderophores (25 μM of NH_2_OH equivalents) while strain A41C3 produced mainly catecholate-type siderophores (70 μM of DHBA equivalents) and showed more affinity to copper or to iron, respectively.

**FIGURE 2:**
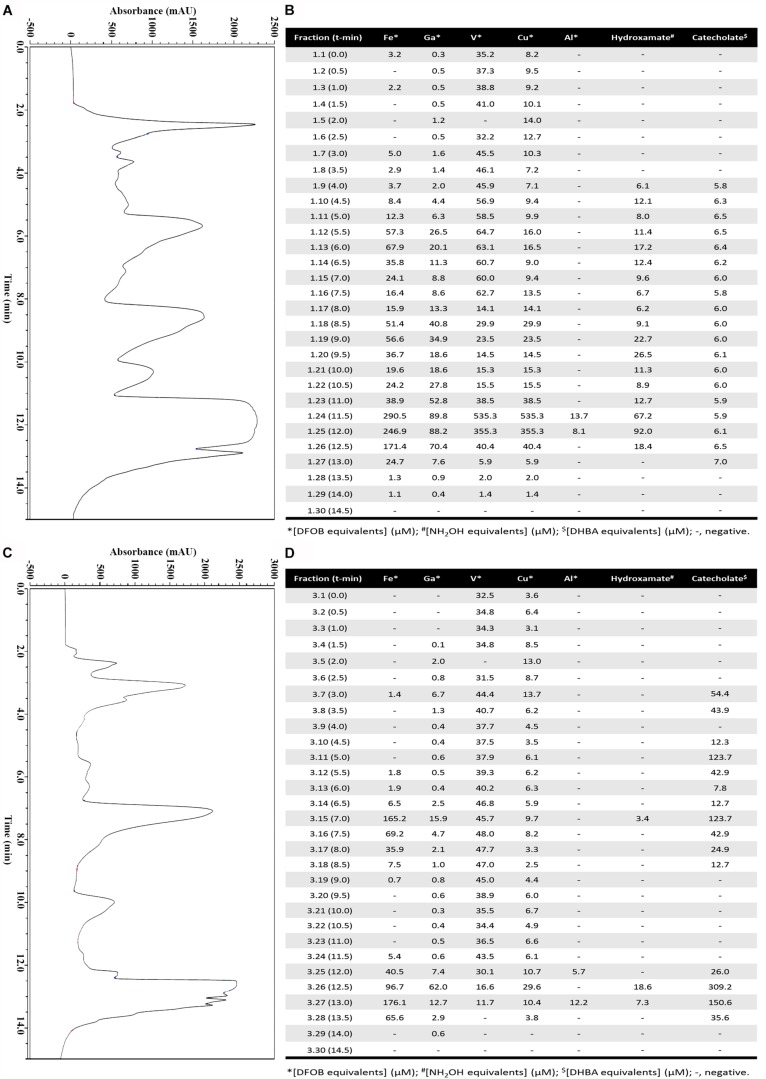
Purification and characterization of the siderophores in supernatant of strains Arv20#4.1 **(A,B)** and A41C3 **(C,D)**. **(A,C)** The siderophores were enriched by HPLC using a C18-RP column (1 ml; Thermo, Auqastar) equilibrated in 28% methanol (buffer **A**) and 72% ddH_2_O with 0.1% TFA (buffer **B**). The elution from the column was performed as described and after 8 min, a 4-min linear gradient 28–95% buffer **A** followed by 3 min 95% buffer **A** was applied before going back to initial conditions (total time of elution 15 min at 1 ml/min flow rate). **(B,D)** Characterization of 30 fractions for Fe, Ga, Cu, V, and Al-CAS activities and quantitation of hydroxamate and catecholate-type siderophores. The best supernatant HPLC fraction of strain Arv20#4.1 containing hydroxamate-type siderophores (92 μM of NH_2_OH equivalents) most actively chelated Cu **(B)**. The best supernatant HPLC fraction of strain A41C3 contains catecholate-type siderophores (309 μM of DHBA equivalents) with higher affinity for Fe **(D)**.

After three days, the growth medium of strain Arv20#4.1 had a pH of 9 while, that of A41C3 a pH of 5. At this moment siderophore comprising supernatants were harvested and processed by ion exchange material to obtain an enriched siderophore pool for each strain. After concentration by evaporation, siderophores were enriched by HPLC and 30 fractions per strain were obtained (Figure 2). Both strains showed ability to produce hydroxamate and catecholate-type siderophores but with different concentrations. The best metal affinity was identified in an HPLC fraction (1.25) of supernatant of strain Arv20#4.1 for a hydroxamate-type siderophore (92 μM of NH_2_OH equivalents) with higher affinity for Cu (Figure 2B). On the other hand, the best supernatant HPLC fraction (3.26) of strain A41C3 contained a catecholate-type siderophore (309 μM of DHBA equivalents) with higher affinity for Fe (Figure 2D).

### Siderophore Identification by Mass Spectrometry

The collected compounds in HPLC fractionations were characterized by using LC-MS. Fragment spectra were analyzed by molecular networking, which allows sorting of spectra according to their similarity that is ultimately based on the structural similarity of molecules analyzed (Wang et al., 2016). Guided by the genome-based biosynthetic potential mass spectrometric data were compared with literature and databases to identify the enriched molecules (Senges et al., 2018).

**FIGURE 3:**
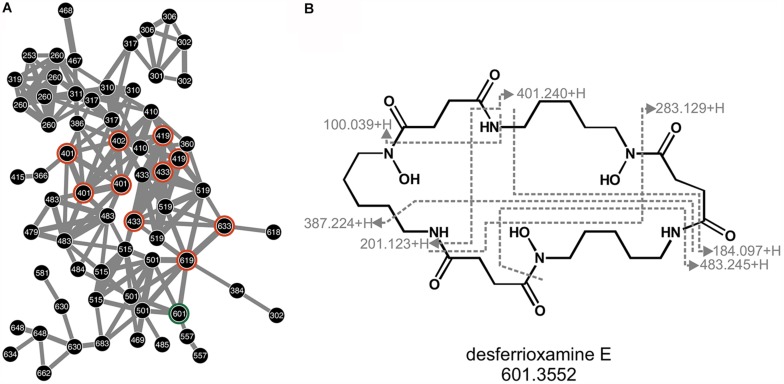
Desferrioxamines of strain Arv20#4.1. Subnetwork of desferrioxamines as produced by Arv20#4.1 and detected in positive mode **(A)**. Nodes are labeled with corresponding m/z values. Highlighted in orange are nodes which resemble identified desferrioxamines. As this network is not dereplicated, some desferrioxamines are represented by several nodes. Marked in green is desferrioxamine E, for which the corresponding fragment spectrum was mapped onto the molecular structure **(B)**. Given are the m/z of fragments as measured in positive mode.

As indicated by its desferrioxamine biosynthetic gene cluster, Arv20#4.1 produces a set of desferrioxamines (Figure 3A). These were detected in both, positive and negative mode from the LC-MS procedure (Supplementary Figure S4). As the eponymous compound for this molecular subnetwork the hydroxamate siderophore desferrioxamine E was identified by comparison with reference spectra described in Senges et al. (2018) and through manual mapping of the fragment spectrum onto the molecular structure (Figure 3B). As sortation of fragment spectra by molecular networking allows sortation into structurally related families, further compounds in the desferrioxamine subnetwork are likely desferrioxamine E analog. This is supported by five further desferrioxamine-like siderophores, identified by their complete masses (Supplementary Table S1).

Of the 421 and 1,034 nodes in the molecular network of A41C3, originating from positive and negative mode respectively, none could be identified. One fragment spectrum showed similarity to a database entry of a desferrioxamine, but its characteristics (parent and fragment masses) were unlike those of typical desferrioxamines. Iron chelating and biological activity are likely to originate from different, yet unknown compounds. Respectively, an overview on siderophore activity of fractions with corresponding signals in mass spectrometry are provided in Figure 2 and Supplementary Table S2.

### *C. elegans* Attracted by Siderophore-Producing Bacteria

Both bacteria, after 24 h growth, showed higher OD_600_ in the M9 growth media supplemented with iron than in low-iron medium. This indicates iron limitation creates stress and should induce a metabolic response as shown above for siderophore production. Strain Arv20#4.1 reached an OD_600_ of 2.06 when growth medium was supplemented with iron and 0.84 when growth medium was deprived of iron. In the same way, strain A41C3 showed OD_600_ of 2.19 when growth medium was supplemented with iron and 0.54 when growth medium was deprived of iron.

**FIGURE 4:**
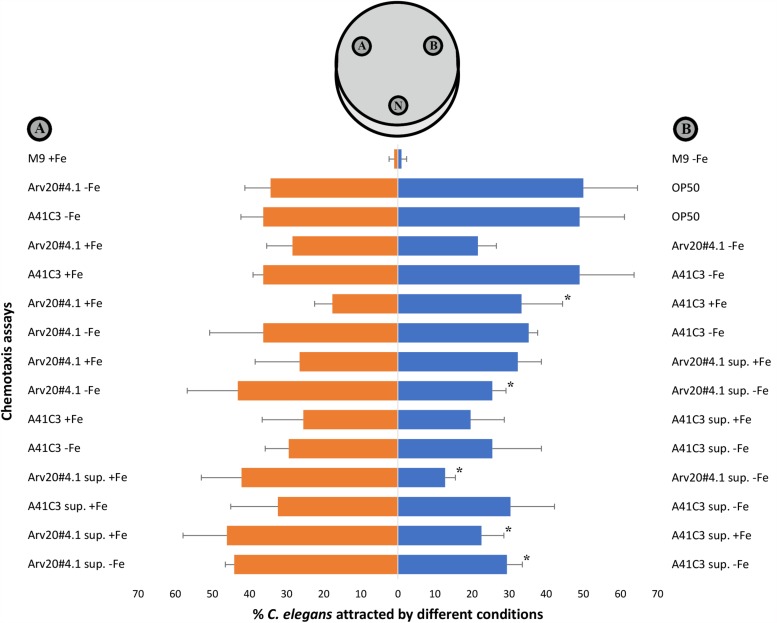
Attraction of *C. elegans* N2 by strains Arv20#4.1 and A41C3 and by their supernatants. The nematodes were significantly attracted by strain A41C3 (+Fe) instead Arv20#4.1 (+Fe) that was not significantly attracted in assay of both bacterial strains without iron supplementation in M9 broth media. The supernatant of Arv20#4.1 (+ Fe) showed significant attraction of nematodes when compared with supernatant of Arv20#4.1 (–Fe) or in the presence of supernatant of A41C3 (+ Fe). On the other hand, supernatant of Arv20#4.1 (–Fe) showed significant attraction of *C. elegans* N2 when compared with supernatant of A41C3 (–Fe). M9 broth media supplemented or not with iron did not attracted nematodes by itself. The *E. coli* OP50 was used as control and nematodes showed preference for their regular food source in comparison with both strains but without statistical significance. N, application spot of nematodes; **(A,B)**, application spots of strains, supernatants or M9 medium. ^∗^Indicate significantly different percentages of attraction (*p* < 0.05) between treatments. sup., abbreviation of supernatant. +Fe, growth medium supplemented with iron. –Fe, growth medium without supplementation of iron.

*C. elegans* N2 was attracted by strains Arv20#4.1 and A41C3 and by their supernatants (Figure 4). The M9 broth media supplemented or not supplemented with iron did not attracted nematodes by itself. The *E. coli* OP50 was used as control and nematodes showed preference for their regular food source in comparison with both strains but without statistical significance. The nematodes were significantly attracted by strain A41C3 (+ Fe) compared to Arv20#4.1 (+ Fe), but no significant attraction of nematodes was observed when both strains were grown in M9 broth medium without iron supplementation. These differences might be due to differences in the metabolites produced and maximum cell density reached (OD_600_). The supernatant of Arv20#4.1 with iron in the growth medium showed significant attraction of nematodes when compared with same strain supernatant without iron. The same was observed when supernatant of Arv20#4.1 (+ Fe) compared with supernatant of A41C3 supplemented with iron (Figure 4). On the other hand, supernatant of Arv20#4.1 without iron showed significant attraction of nematodes when compared with supernatant of A41C3 without supplementation of iron (Figure 4).

### Nematicidal Activity

The herein investigated bacteria and their supernatants did not show nematicidal activity against *C. elegans* N2 evaluated at 24 h, 48h, and 72h incubation.

**FIGURE 5:**
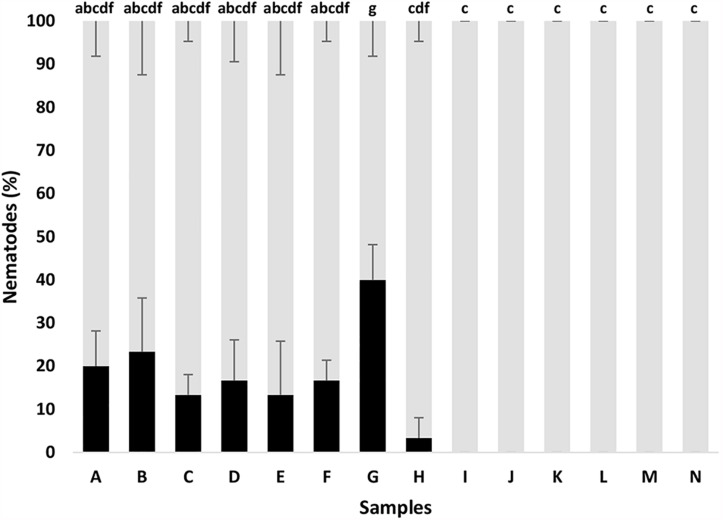
*Bursaphelenchus xylophilus* dead (black) and alive (gray) after different *in vitro* treatments with bacterial supernatant and fractions containing siderophores, after 24 h incubation. Fractions, mentioned in Figure 2, with siderophores resuspended in 0.1 M NaCl. (A) HPLC fractions 1.12 + 1.13; (B) 1.17 + 1.18 + 1.19; (C) HPLC fractions 1.21 + 1.22; (D) HPLC fractions 1.23 + 1.24 + 1.25 + 1.26; (E) HPLC fractions 3.14 + 3.15 + 3.16; (F) HPLC fractions (3.20 + 3.21); (G) HPLC fractions 3.25 + 3.26 + 3.27 + 3.28; (H) NaCl 0.1 M; (I) Arv20#4.1 supernatant (growth in CAA medium); (J) A41C3 supernatant (growth in CAA medium); (K) Arv20#4.1 supernatant (growth in M9 medium); (L) A41C3 supernatant (growth in M9 medium); (M) CAA medium; and (N) M9 medium. Different letters above the bars indicate significantly different percentages of mortality (*p* < 0.05) between treatments.

In the same line, the bacterial supernatants and fractions of both strains containing siderophores were tested against *B. xylophilus*. It was possible to observe significant nematicidal activity for HPLC fractions of the samples A-G against *B. xylophilus* PtAS18 (Figure 5). The sample G, containing HPLC fractions 3.25 + 3.26 + 3.27 + 3.28, showed the strongest nematicidal activity that includes combination of HPLC fractions 25, 26, 27, 28 (equal ratio) of the strain A41C3. The compounds present in these HPLC fractions, with nematicidal activity, showed similarity to a number of database entries with respect to the MS analysis. However, an identification was impossible so far. Therefore, the amounts were too little to obtain a relevant fragmentation pattern or even NMR data after iron removing.

## Discussion

Bacteria are important in the ecosystem of *P. pinaster* trees as they are involved in key activities and interactions such as production of proteases, lipases, cellulases, secretion of metabolites for signaling and auxins (Proença et al., 2010, 2017a,b; Vicente et al., 2012). They are further involved in solubilization of phosphate and zinc and mobilization of metals and metalloids by the production of siderophores (Proença et al., 2010, 2017a,b). Among those activities, (secondary) metabolite production and release by microbes represents an important trigger in the ecosystems. This is often a response towards a limitation of resources within such an ecosystem. Here, we focused especially on metallophores produced under metal (iron) limitation and known to have species overlapping activities (Hider and Kong, 2010; Wiche et al., 2017; Senges et al., 2018). Metallophores are known to be part of the arsenal of plant-growth promoting (rhizo)bacteria and to interact with other microorganisms, nematodes, higher animals, and plants within the ecosystem (Mercado-Blanco et al., 2018). Metabolites like siderophores play important roles in ecosystems to chelate metallic micronutrients essential for microorganisms and plants. However, siderophores have been recognized to have a key role in virulence of pathogenic organisms (Soutar and Stavrinides, 2018) and thus its screening is important.

Species belonging to the family *Enterobacteriaceae* are known to grow in soil next to plants, to engage in plant-microbe interactions, and thus are part of a complex network in the rhizosphere. Strains of the genus *Rouxiella* have been isolated from peat bog soil (Le Fléche-Matéos et al., 2017) and are recognized to produce biosurfactants (Kügler et al., 2015). Strains belonging to both genera *Erwinia* and *Pantoea* have been demonstrated to produce hydroxamate siderophore desferrioxamine E among others that supports the iron acquisition by the strains. This ability favors host colonization and it is recognized as a factor of pathogenicity (Dellagi et al., 2009).

Here, we found that the endophytic *Erwinia* strain A41C3 produces a catecholate-type siderophore based on analytical methods and bioinformatics genome annotation pipeline. The latter hints towards turnerbactin but this was not verified by LC-MS/MS and thus, these compounds remain enigmatic and await structural identification. *Erwinia* species are known to produce achromobactin or aerobactin (Soutar and Stavrinides, 2018), two hydroxamate-type siderophores, that have not yet been described to produce catecholate-type metallophores like turnerbactin-related compounds. This will be investigated in more detail in the near future.

In this study, we found that PWN-carried *Rouxiella* strain Arv20#4.1 produces desferrioxamine-like compounds based on analytical methods and also predicted from the genome (Figure 3 and Supplementary Figure S2). This was verified by LC-MS/MS confirming the identification of desferrioxamine-like structures, with desferrioxamine E (DFOE) as major metabolite. This is the first time that siderophore production was described in more detail by a strain of the genus *Rouxiella* and the respective siderophore was identified as DFOE. The HPLC-enriched fraction of DFOE and related molecules (Figure 2) showed affinity to various metals and metalloids as described previously for other desferrioxamines obtained from soil bacteria (Mehnert et al., 2017; Wiche et al., 2017; Schwabe et al., 2018).

As described above both strains, A41C3 and Arv20#4.1, produced different siderophores which have different properties. As various metals and metalloids may play a role in the rhizosphere ecosystem it is of interest to verify the capability of the siderophores to bind various types of ions (Mehnert et al., 2017; Wiche et al., 2017; Schwabe et al., 2018). Therefore, we decided to choose a selection of ions which can form a CAS-complex and thus allow to show and to discriminate affinity of siderophores for Fe, Ga, Cu, V, and Al (Figure 2). The siderophore of strain Arv20#4.1 is a hydroxamate-type siderophore and has a high affinity for Cu. The one of strain A41C3 is a catecholate-type siderophore and has a high affinity for Fe.

*Caenorhabditis elegans* N2 were attracted by both strains and by their supernatants. This attraction of this nematode through different bacterial species has been recognized in several studies with other bacterial strains (Choi et al., 2016). No nematicidal activity against *C. elegans* was demonstrated by either species, by bacterial supernatants, or by HPLC fractions containing siderophores. Monitoring food behavior of *C. elegans* is a simple approach to identify potential bacterial virulence (Lewenza et al., 2014). Considering that the biological role of siderophores is to ensure the acquisition of iron necessary for metabolism, their production is expected to be lower in bacteria growing in iron-supplemented media. This was the case, however, the nematode behavior was similar in both supernatants. *C. elegans* preferred bacterial supernatants to bacterial cells, independently of the bacterial growth conditions. This behavior is in congruence with the fact that bacterial supernatants and siderophores do not kill *C. elegans* and may be used by the nematode as food sources. The type of siderophores, and probably the concentration, might be crucial for nematicidal activity as it was demonstrated in pathogenicity of *P. aeruginosa* by its siderophore pyoverdin (Kirienko et al., 2013). On the other hand, the nematode behavior seems to indicate that *Rouxiella* sp. Arv20#4.1 is more virulent than *Erwinia* sp. A41C3, using virulence factors different from siderophores. Both bacterial species are *gamma-proteobacteria* reported to be pathogenic (Paiva et al., 2013).

The effect of enriched siderophore fractions on *B. xylophilus* behavior was evaluated for the first time. The isolate Arv20#4.1 originating from PWN did produce mainly desferrioxamines which had no effect on *B. xylophilus*. Thus it might be reasoned that desferrioxamines interfere with the mobility of metals but do not affect other organisms of this ecosystem to a large extent. However, this nematode showed to be highly susceptible to supernatants containing siderophores and other metabolites produced by the endophytic *Erwinia* sp. A41C3 under iron limitation. Here, the highest nematicidal activity was determined from the fractions comprising highest siderophore titer, respectively. This seems to be a clear correlation and needs to be elucidated with pure compounds in more detail. Siderophores produced by entomopathogenic bacteria (Hirschmann et al., 2017), endophytic plant growth-promoting bacteria (Tashi-Oshnoei et al., 2017), and rhizospheric bacteria (Khan et al., 2016) have been related with the pathogenic activity of these bacteria against insects, plant pathogenic bacteria and root-knot nematode, respectively. The role of this *Erwinia* strain when part of the endophytic bacterial community needs to be explored. In spite of that, their metabolites produced under iron limitation should be investigated further as they might prove useful in biocontrol *B. xylophilus* and management of Pine Wilt Disease.

## Data Availability Statement

The Whole Genome Shotgun project of strains Arv20#4.1 and A41C3 has been deposited at ENA under the accession numbers RQVU00000000 and RQVV00000000, respectively. The 16S rRNA gene sequences of the isolates have been previously deposited in the NCBI GenBank database under the accession numbers KF214948 (Proença et al., 2014) and KJ654833 (Proença et al., 2017a). Accession links for metabolomic data sets are provided at: positive mode (http://gnps.ucsd.edu/ProteoSAFe/status.jsp?task=bbd4f668b6224bd381617001be917381); negative mode (http://gnps.ucsd.edu/ProteoSAFe/status.jsp?task=e3009a 90a0954791ae9b747e727c6515).

## Author Contributions

DP, PM, and DT designed the experiments and wrote the manuscript. DP did all the experiments. TH did the genome sequencing and analysis. CS performed the mass spectrometry analysis. DT and PM contributed with reagents and laboratory conditions. JB provided input to the manuscript. All authors approved the manuscript.

## Conflict of Interest

The authors declare that the research was conducted in the absence of any commercial or financial relationships that could be construed as a potential conflict of interest.

## Footnotes

1 http://eztaxon-e.ezbiocloud.net/

2 http://www.arb-silva.de/projects/living-tree/

3 https://usegalaxy.eu

4 http://www.ncbi.nlm.nih.gov/genomes/static/Pipeline.html
