# Supplementary material for: Bacterial Metabolites Produced Under Iron Limitation Kill Pinewood Nematode and Attract Caenorhabditis elegans
Source: Front Microbiol. 2019 Sep 19;10:2166. doi: 10.3389/fmicb.2019.02166 (PMC6761702; doi:10.3389/fmicb.2019.02166)
Supplement: Supplementary file 1 [file Data_Sheet_1.PDF]

# *Supplementary Material*

## 1.1 Supplementary Figures

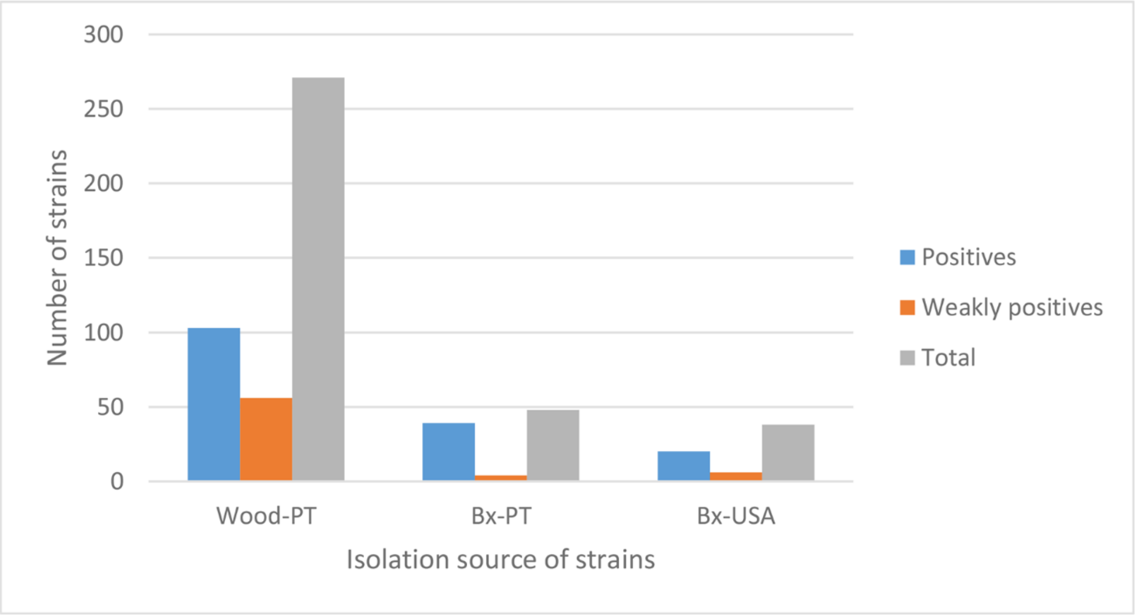


**Supplementary Figure 1.** Siderophore production on CAS agar of endophytic, wood colonizing bacteria, isolated in Portugal (Wood-PT) and of *Bursaphelenchus xylophilus*-carried bacteria isolated in Portugal (Bx-PT) and USA (Bx-USA). In total, 103 endophytic strains (38.0 %), 39 strains carried by *B. xylophilus* from Portugal (81.2 %) and 20 strains carried by *B. xylophilus* from USA (52.6 %) were positive for siderophore production on CAS agar. Blue bars, positive strains for siderophore production on CAS agar plates; Orange bars, weakly positive strains for siderophore production on CAS agar plates; Gray bars, total of bacterial strains tested. Bx, *B. xylophilus*. PT, Portugal. USA, United States of America.

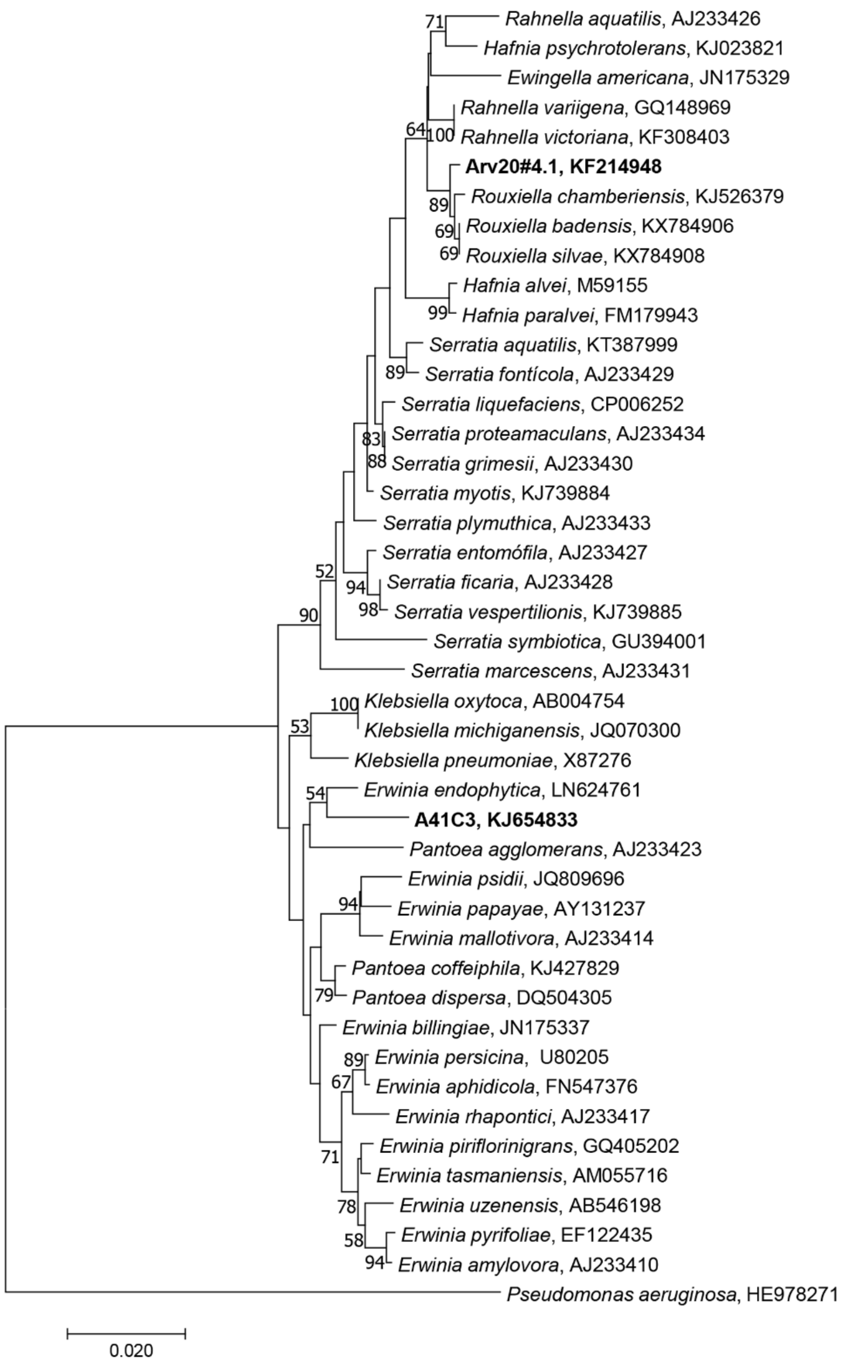


**Supplementary Figure 2.** Phylogenetic dendrogram based on a comparison of the 16S rRNA gene sequence of strains Arv20#4.1 and A41C3, and representatives of the closest *Enterobacteriaceae* type strains. The tree was created using the neighbor-joining method. The numbers on the tree indicate the percentages of bootstrap sampling, derived from 1,000 replications; values below 50% are not shown. The isolates characterized in this study are indicated in bold. Scale bar, 2 inferred nucleotide substitution per 100 nucleotides.

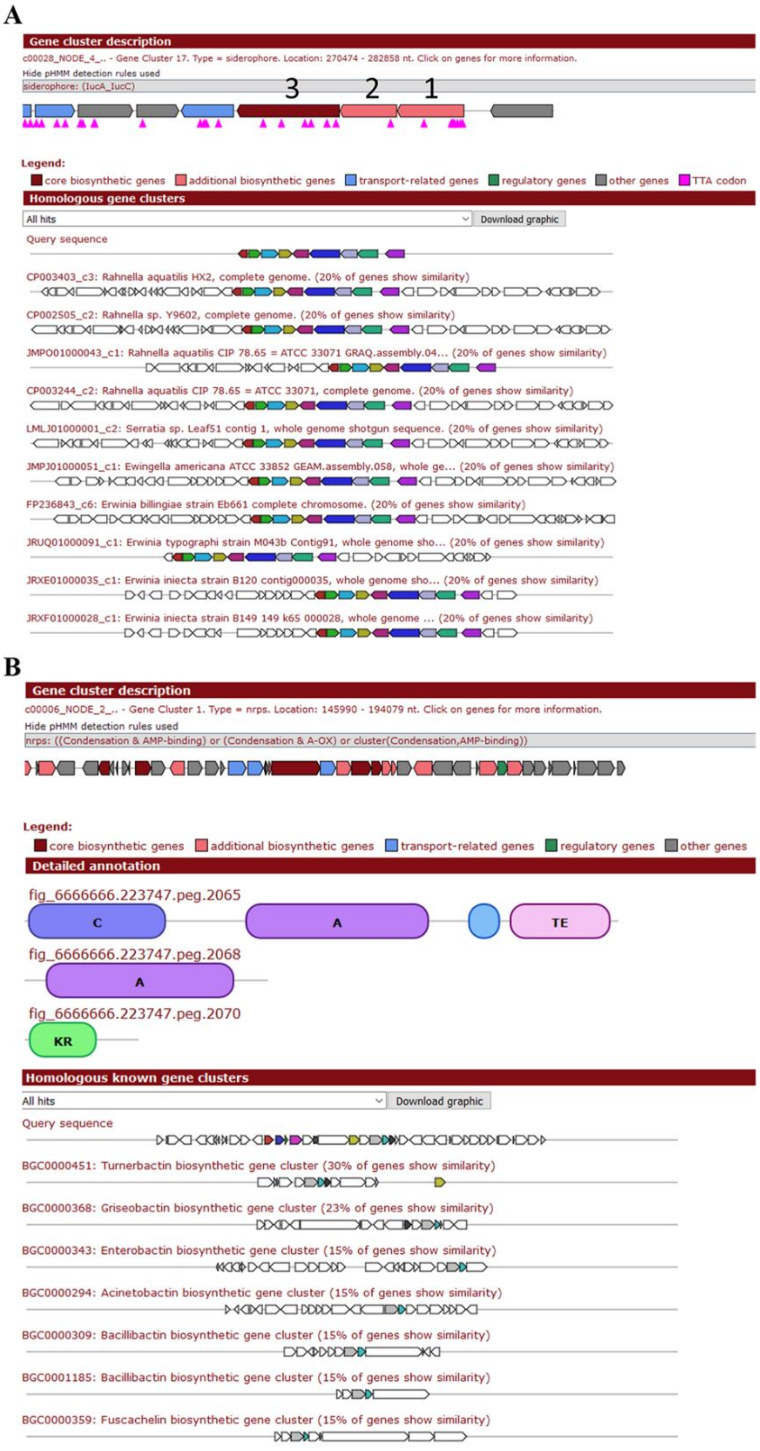


**Supplementary Figure 3.** Genetic organization of siderophores biosynthetic gene clusters from the genomes of the strains Arv20#4.1 (A) and A41C3 (B). Both strains showed similarities with siderophore biosynthetic gene clusters identified from other strains. Strain Arv20#4.1 showed the necessary genes to produce the necessary enzymes involved in the production of hydroxamate-type siderophores: 1- decarboxylase, pyridoxal-dependent, 2- lysine/ornithine N-monooxygenase, 3- putative siderophore biosynthesis protein - GNAT family N-acetyltransferase. Genome of strain A41C3 showed potential to produce new siderophore(s). (Figure adapted from results obtained from ANTISMASH website).

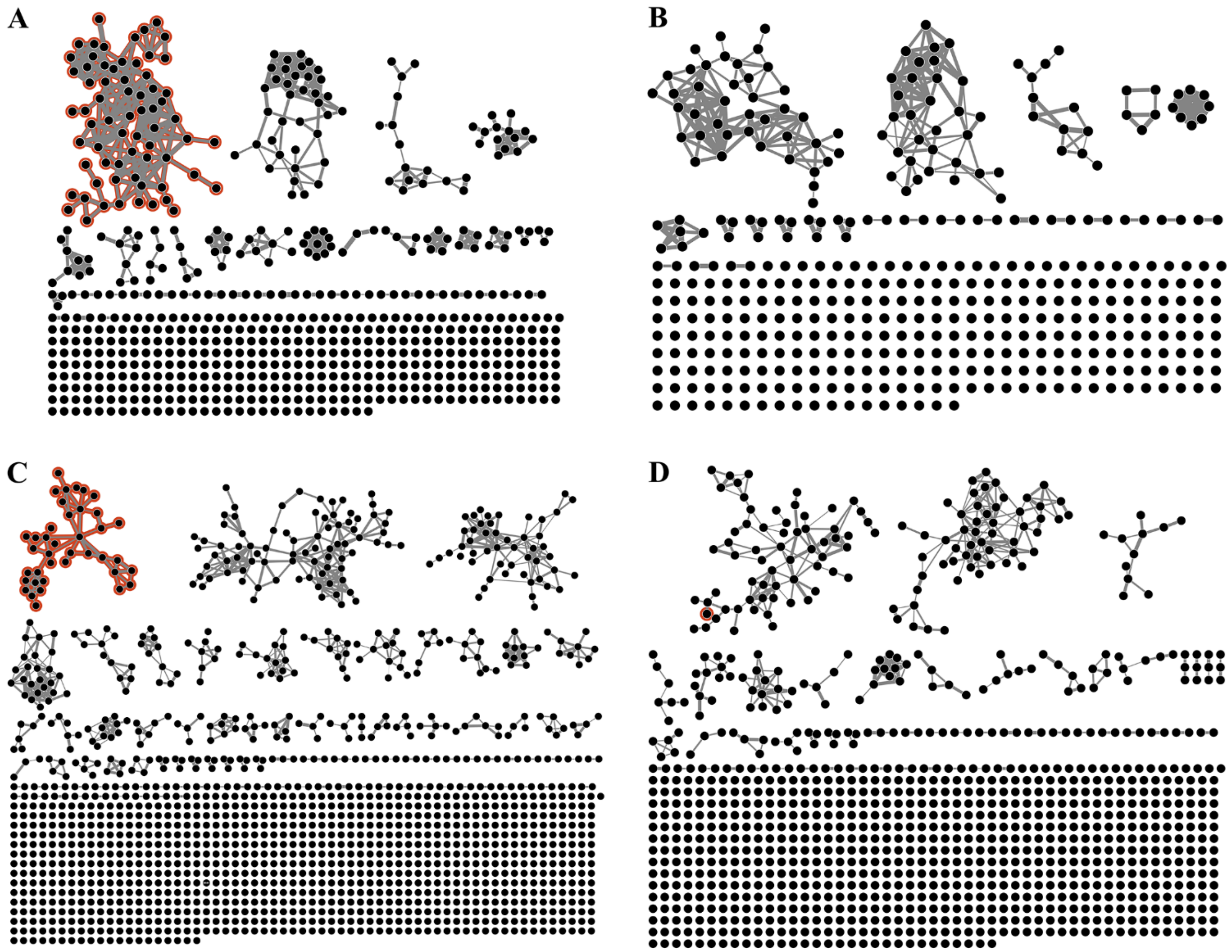

**Supplementary Figure 4.** Molecular network of metabolites produced by strains Arv20#4.1 and A41C3. Nodes resemble metabolites as measured in positive (A and B) and negative (C and D) mode, respectively. Nodes are connected if the cosine similarity of fragment spectra is ≥ 0.7 (line thickness reflects similarity score). Highlighted in orange are the subnetworks corresponding to desferrioxamine siderophores of strain Arv20#4.1 (A and C) and of strain A41C3 (D).

## 1.2 Supplementary Tables

**Table S1.** Identification of Desferrioxamine like molecules obtained from strain Arv20#4.1 by MS. Obtained m/z values are compared with theoretical values.

| Compound | theoretical value | | measured value | | Δppm | |
|---|---|---|---|---|---|---|
| | m/z + $H^+$ | m/z - $H^+$ | m/z + $H^+$ | m/z - $H^+$ | + | - |
| Desferrioxamine E | 601.3561 | 599.3405 | 601.3552 | 599.3398 | 1.50 | 1.17 |
| Desferrioxamine G1 | 619.3667 | 617.351 | 619.3663 | 617.3522 | 0.65 | 1.94 |
| Bisucaberin | 401.24 | 399.2244 | 401.2412 | 399.2241 | 2.99 | 0.75 |
| Bisucaberin B | 419.2506 | 417.2349 | 419.2489 | 417.2336 | 4.05 | 3.12 |
| | | | | | | |
| Bisu-02 * | 433.2662 | 431.2506 | 433.2656 | n.d. | 1.38 | n.d. |
| Desf-01 * | 633.3817 | 631.3667 | 633.3832 | 631.3678 | 2.37 | 1.74 |

n.d. = not detected

*Compounds which had been described previously by Senges et al. 2018 and are so far only predicted structures.

**Table S2.** Prominent massed detected by LC-MS from relevant fractions of strain A41C3. Elution profiles of HPLC fractions with siderophore activity (Figure 2) and those which showed nematicidal activity (25 to 28) are shown for positive and negative mode.

| **positive mode** | | | **signals of HPLC fractions (numbered according to Fig. 2)** | | | | | | |
|---|---|---|---|---|---|---|---|---|---|
| **Parent Mass** | **RT-Mean** | **RT-StdErr** | **24** | **25** | **26** | **27** | **28** | **29** | **Chromatogram** |
| 697.01 | 824.8794 | 36.02081 | 0 | 0 | 0 | 0.696969 | 0.303030 | 0 | 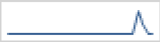 |
| 501.02 | 732.3644 | 45.61451 | 0 | 0 | 0.296296 | 0.370370 | 0.333333 | 0 | 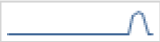 |
| 716.14 | 793.0768 | 25.64382 | 0 | 0.4 | 0.6 | 0 | 0 | 0 | 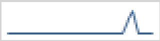 |
| 719.22 | 780.0502 | 24.37521 | 0 | 0.236842 | 0.763157 | 0 | 0 | 0 | 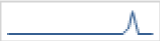 |
| 685.34 | 799.4890 | 76.42420 | 0 | 0.196078 | 0.215686 | 0.411764 | 0.176470 | 0 | 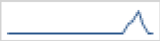 |
| 688.55 | 725.1168 | 4.191811 | 0 | 0.270270 | 0.432432 | 0.297297 | 0 | 0 | 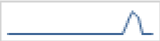 |
| 686.20 | 784.5548 | 72.24246 | 0 | 0.24 | 0.14 | 0.42 | 0.2 | 0 | 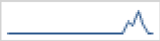 |
| 702.97 | 744.0640 | 54.01544 | 0 | 0.465517 | 0.5 | 0.034482 | 0 | 0 | 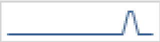 |

| **negative mode** | | | **signals of HPLC fractions (numbered according to Fig. 2)** | | | | | | |
|---|---|---|---|---|---|---|---|---|---|
| **Parent Mass** | **RT-Mean** | **RT-StdErr** | **24** | **25** | **26** | **27** | **28** | **29** | **Chromatogram** |
| 681.79 | 775.6771 | 55.82194 | 0 | 0.166666 | 0.5 | 0.166666 | 0.166666 | 0 | 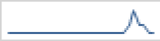 |
| 679.19 | 706.6628 | 28.45990 | 0.093023 | 0.232558 | 0.209302 | 0.209302 | 0.046511 | 0 | 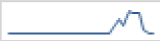 |
| 360.01 | 708.3951 | 3.034697 | 0 | 0 | 0 | 1 | 0 | 0 | 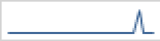 |
| 361.08 | 809.9292 | 2.750614 | 0 | 0.333333 | 0.666666 | 0 | 0 | 0 | 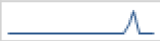 |
| 709.43 | 852.6492 | 8.041743 | 0 | 0 | 0 | 0.466666 | 0.533333 | 0 | 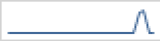 |
| 695.21 | 798.1454 | 8.944550 | 0 | 0 | 0 | 0.5 | 0.5 | 0 | 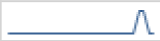 |
| 363.17 | 896.6154 | 9.734801 | 0 | 0 | 0 | 0 | 0.533333 | 0.466666 | 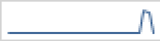 |
| 403.13 | 919.1174 | 6.290099 | 0 | 0 | 0 | 0 | 0.857142 | 0.142857 | 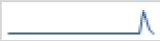 |
| 319.18 | 912.083 | 3.912039 | 0 | 0 | 0 | 0 | 0.666666 | 0.333333 | 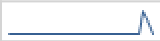 |
| 342.53 | 690.9268 | 3.012472 | 0 | 0.333333 | 0.666666 | 0 | 0 | 0 | 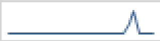 |
| 364.52 | 681.7944 | 4.397904 | 0 | 0.285714 | 0.357142 | 0.357142 | 0 | 0 | 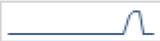 |
| 355.52 | 727.8247 | 11.02184 | 0 | 0 | 0 | 0.416666 | 0.333333 | 0.25 | 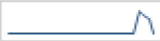 |
| 681.23 | 788.3335 | 5.093642 | 0 | 0 | 0.15 | 0.4 | 0.45 | 0 | 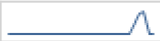 |
| 417.12 | 764.2607 | 3.187951 | 0 | 0.058823 | 0.529411 | 0.411764 | 0 | 0 | 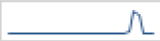 |
| 697.19 | 754.4464 | 76.08200 | 0 | 0.177777 | 0.222222 | 0.377777 | 0.222222 | 0 | 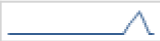 |
| 684.12 | 686.1684 | 43.44985 | 0.037037 | 0.296296 | 0.296296 | 0.370370 | 0 | 0 | 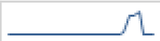 |
| 875.22 | 753.676 | 3.535792 | 0 | 0 | 0.529411 | 0.470588 | 0 | 0 | 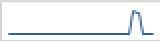 |
| 690.11 | 814.363 | 4.135068 | 0 | 0 | 0 | 0.5 | 0.5 | 0 | 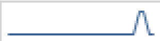 |
| 445.08 | 754.5411 | 4.212591 | 0 | 0 | 0 | 0.5 | 0.5 | 0 | 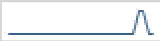 |
| 716.15 | 772.6373 | 7.699298 | 0 | 0.344827 | 0.310344 | 0.344827 | 0 | 0 | 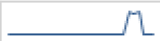 |
| 700.12 | 726.5115 | 13.99120 | 0 | 0.333333 | 0.370370 | 0.296296 | 0 | 0 | 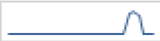 |
| 802.12 | 901.6801 | 4.052763 | 0 | 0 | 0 | 0.428571 | 0.571428 | 0 | 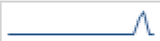 |
| 668.13 | 808.2528 | 9.399822 | 0 | 0 | 0.307692 | 0.346153 | 0.346153 | 0 | 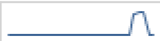 |
| 686.15 | 680.4915 | 24.80066 | 0.020408 | 0.204081 | 0.183673 | 0.387755 | 0.204081 | 0 | 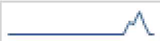 |
